# Performance of GPT-4o and DeepSeek-R1 in the Polish Infectious Diseases Specialty Exam

**DOI:** 10.7759/cureus.82870

**Published:** 2025-04-23

**Authors:** Zuzanna Błecha, Dawid Jasiński, Aleksander Jaworski, Ada Latkowska, Wojciech Jaworski, Oliwia Sysło, Nikola Rubik, Izabela Jastrzebska, Konrad Haraziński, Weronika Goliat, Maksym Gmur, Michal Gajewski, Barbara Sławińska, Nicole Maryniak

**Affiliations:** 1 Faculty of Medicine, Medical University of Silesia in Katowice, Katowice, POL; 2 Department of Paediatric Cardiology, Saint John Paul II Upper Silesian Child Health Centre, Public Clinical Hospital no.6 of the Medical University of Silesia in Katowice, Katowice, POL; 3 Plastic Surgery, Specialist Medical Center, Polanica-Zdrój, POL; 4 Faculty of Medicine, Wroclaw Medical University, Wrocław, POL; 5 Department of Children’s Developmental Defects Surgery and Traumatology SUM (Medical University of Silesia) Scientific Club, Medical University of Silesia in Katowice, Katowice, POL; 6 Faculty of Medicine, Academy of Silesia, Katowice, POL; 7 Medicine, Academy of Silesia, Katowice, POL; 8 Internal Medicine, Międzyleski Specialized Hospital in Warsaw, Warsaw, POL; 9 Medicine, Medical University of Silesia in Katowice, Katowice, POL; 10 Medicine, Provincial Hospital in Poznań, Poznań, POL; 11 Medicine, Zagłębiowski Oncology Center in Dąbrowa Górnicza, Dąbrowa Górnicza, POL

**Keywords:** #artificial intelligence, #chatgpt, deepseek, #final medical examination, infectious disease medicine, #machine learning, #medical professionals, #medical students

## Abstract

Background

The past few years have been a time of rapid development in artificial intelligence (AI) and its implementation across numerous fields. This study aimed to compare the performance of GPT-4o (OpenAI, San Francisco, CA, USA) and DeepSeek-R1 (DeepSeek AI, Zhejiang, China) on the Polish specialty examination in infectious diseases.

Materials and methods

The study was conducted from April 1 to April 4, 2025, using the Autumn 2024 Polish specialty examination in infectious diseases. The examination comprised 120 questions, each presenting five answer options, with only one correct choice. The Center for Medical Education (CEM) in Łódź, Poland decided to withdraw one question due to the absence of a definitive correct answer and inconsistency with up-to-date clinical guidelines. Furthermore, the questions were classified as either 'clinical cases' or 'other' to enable a more in-depth evaluation of the potential of artificial intelligence in real-world clinical practice. The accuracy of the responses was verified using the official answer key approved by the CEM. To assess the accuracy and confidence level of the responses provided by GPT-4o and DeepSeek-R1, statistical methods were employed, including Pearson's χ^2^ test, and Mann-Whitney U test.

Results

GPT-4o correctly answered 85 out of 199 questions (71.43%) while DeepSeek-R1 answered correctly 88 out of 199 questions (73.85%). A minimum of 72 (60.5%) correct responses is required to pass the examination. No statistically significant difference was observed between responses to 'clinical case' questions and 'other' questions for either AI model. For both AI models, a statistically significant difference was observed in the confidence levels between correct and incorrect answers, with higher confidence reported for correctly answered questions and lower confidence for incorrectly answered ones.

Conclusions

Both GPT-4o and DeepSeek-R1 demonstrated the ability to pass the Polish specialty examination in infectious diseases, suggesting their potential as educational tools. Additionally, it is noteworthy that DeepSeek-R1 achieved a performance comparable to GPT-4o, despite being a much newer model on the market and, according to available data, having been developed at significantly lower cost.

## Introduction

Recent years have seen significant advancements in artificial intelligence (AI) and increasing efforts to apply it across various fields, including medicine [1]. Among the AI models that have recently gained widespread attention and demonstrated rapid advancement is ChatGPT, developed by OpenAI (San Francisco, CA, USA). [2]. Since its launch in November 2022, there has been a steady and significant increase in interest in using ChatGPT, currently reaching 400 million weekly users (based on data from February 2025). OpenAI’s goal is to grow its user base to one billion by the end of 2025. Also based on data from February 2025, ChatGPT ranks as the eighth most visited website, surpassing platforms owned by companies such as Amazon and Netflix. Direct access remains the dominant source of traffic to ChatGPT, representing 79.77% of all user visits. It is also worth noting that individuals under the age of 24 represent more than 45% of all ChatGPT users [3].

On January 20, 2025, DeepSeek AI (Zhejiang, China), a Chinese tech company, released DeepSeek-R1 - a partially open-source reasoning model intended to rival OpenAI’s flagship Large Language Model (LLM), o1. Unlike OpenAI’s ChatGPT o1, which is available only through a paid ChatGPT subscription, DeepSeek-R1 is freely accessible to users [4,5]. Notably, the development costs of DeepSeek-R1 amounted to six million United States dollars (USD), which is significantly lower compared to the 100 million USD reportedly spent on OpenAI's GPT-4 in 2023. Furthermore, DeepSeek-R1 requires only one-10th of the computational resources needed by OpenAI's model [6]. Following the release of the DeepSeek-R1 chatbot application on January 10, 2025, the application surpassed ChatGPT by January 27 to become the most downloaded free application on the United States App Store. This development was accompanied by an 18% decline in Nvidia’s stock price, as the company’s GPU hardware is utilized in the training of OpenAI’s LLM [7,8].

Over the past few years, numerous studies have been conducted to evaluate the effectiveness of AI models developed by OpenAI. Jaworski et al. conducted a study comparing the ability of GPT-3.5 and GPT-4o to complete the Polish Final Medical Examination (LEK). In the study, GPT-3.5 failed the exam, whereas GPT-4o passed it with a score of 77.5%, which was comparable to the average score achieved by human examinees [9]. In another study, Jaworski et al. demonstrated that GPT-4o is capable of passing the Polish Final Dentistry Examination (LDEK), achieving a score of 70.85% [10]. The capabilities of ChatGPT have also been assessed in specialist medical examinations, including those in cardiology [11], nuclear medicine [12], dermatology [13], and allergology [14]. Due to the relatively recent introduction of the DeepSeek-R1 model, the body of research examining its performance remains limited. Lisle Faray de Paiva et al. conducted a study evaluating the performance of the DeepSeek-R1 model in the United States Medical Licensing Examination (USMLE), where they observed superior effectiveness compared to ChatGPT in Step 1 and Step 2 tasks requiring act-based recall and clinical knowledge retrieval. Nevertheless, at the Step 3 level, where clinical case analysis plays a more prominent role, although DeepSeek-R1's responses remain closer to the correct answers than those of the OpenAI model, its extractive methodology proves insufficient in capturing the nuances of patient management decisions [15].

The aim of our study was to compare two AI models, GPT-4o and DeepSeek-R1, that are freely available to all users, in their performance on the Polish specialty examination in infectious diseases. This examination is administered at the end of a five-year specialization program in infectious diseases and serves as a prerequisite for obtaining the title of medical specialist in this field.

## Materials and methods

The study was conducted from April 1 to April 4, 2025, using the Autumn 2024 Polish specialty examination in infectious diseases, which was randomly selected from previously administred examinations in the questions database of the Center for Medical Education (Centrum Egzaminów Medycznych, CEM) in Łódź, Poland. The selected examination initially consisted of 120 multiple-choice questions, each with one correct answer among five distractors, chosen using random number generator. The CEM decided to withdraw one question due to the absence of a definitive correct answer and inconsistency with up-to-date clinical guidelines.

For the purposes of comprehensive analysis, all questions were categorized as either 'clinical cases,' requiring the selection of the correct answer based on the clinical description of a specific patient, or 'other,' referring to questions related to infectious diseases that were not presented in a case-based format. Two independent researchers conducted the categorization, which was later reviewed and accepted by a third independent researcher. Due to the multi-center nature of the study, the authors communicated remotely using platforms such as Zoom, Microsoft Teams, Facebook Messenger, Google Docs, and email. All sections of the study prepared by the individual groups were reviewed by the other authors, allowing each researcher to contribute to every part of the study using the aforementioned remote communication methods.

Data collection and analysis

Prior to presenting the questions to each AI model, they were familiarized with the examination guidelines, including the number of answer choices and the number of correct answers. Additionally, after each question was submitted to the AI models, they were asked: "On a scale of one to five, how confident did you feel about the question?" This question was posed in order to assess the level of confidence each AI model had in its response to the given question as follows: one - unsure, two - not very sure, three - almost sure, four - very sure, five - completely sure. All interactions with GPT-4o and DeepSeek-R1 were thoroughly documented. In order to maintain consistency with the content of the examination questions, all interactions with the AI models were conducted in Polish. The study was conducted using the GPT-4o and DeepSeek-R1 AI models.

Statistical analysis

The results obtained from each AI model were compared with the correct answers provided by the CEM in Łódź. The evaluation of each AI model's effectiveness involved calculating the percentage of correct responses generated by the AI models. Additionally, an analysis was conducted on the confidence levels associated with both correct and incorrect answers.

Pearson’s chi-square test was employed to evaluate the association between the distribution of correct and incorrect responses and question type. Statistical analyses were conducted using the STATISTICA software package (StatSoft, Tulsa, OK, USA). In addition, the Mann-Whitney U test was applied to compare the confidence levels between correct and incorrect answers. A p-value of less than 0.05 was considered indicative of statistical significance.

## Results

**Figure 1:**
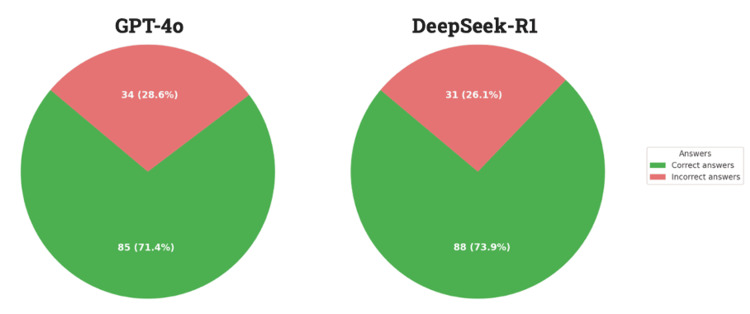
General summary of GPT-4o and DeepSeek-R1. Data are presented as N (%), where N represents the number of correct or incorrect responses and (%) indicates the percentage of all questions they represent.

GPT-4o answered 85 questions correctly (71.43%) and 34 questions incorrectly (28.57%). DeepSeek-R1 answered 88 questions correctly (73.95%) and 31 questions incorrectly (26.05%) (Figure 1). The individual response to each question for each AI model, along with the corresponding confidence level, is presented in Table 1. The full text of each question is publicly available on the CEM website [16]. 

**Table 1:** Table of individual AI model responses and corresponding confidence levels for each question The table presents the responses of both AI models in relation to the correct answers, based on the official answer key published by the Center for Medical Education (Centrum Egzaminów Medycznych, CEM) in Łódź, Poland. Additionally, for each question, the confidence level is also provided, which was determined based on the following prompt: “On a scale of one to five, how confident did you feel about the question?” This question was posed to assess the level of confidence each AI model had in its response to the given question, with the following scale: one – unsure, two – not very sure, three – almost sure, four – very sure, five – completely sure.

Question number	Correct answer	GPT-4o answer	GPT-4o confidence level	Deep-Seek-R1 answer	Deep-Seek-R1 confidence level
1	C	C	5	B	4
2	B	B	5	B	4
3	C	A	4	D	3
4	D	D	5	D	4
5	D	C	4	C	4
6	C	C	5	C	5
7	E	C	5	C	4
8	C	C	5	C	4
9	D	A	4	D	4
10	B	E	5	B	5
11	C	C	5	C	4
12	B	C	4	A	4
13	D	D	5	D	4
14	B	B	5	B	5
15	A	A	5	A	4
16	C	C	5	C	5
17	D	D	4	D	4
18	B	B	5	B	4
19	A	A	4	A	4
20	E	C	5	E	5
21	E	E	5	E	4
22	C	C	5	A	4
23	A	A	5	A	4
24	D	C	4	D	4
25	C	C	4	C	4
26	C	C	5	C	4
27	A	A	5	A	4
28	C	C	5	C	4
29	B	B	5	B	4
30	B	B	5	C	4
31	E	B	5	B	4
32	D	D	5	D	4
33	E	D	5	D	4
34	C	A	4	A	4
35	D	D	5	D	4
36	B	E	4	B	4
37	E	E	5	E	4
38	D	D	5	D	4
39	A	A	5	A	4
40	D	B	5	B	4
41	C	C	5	C	4
42	C	C	5	C	4
43	D	B	5	B	4
44	D	D	5	D	4
45	E	B	5	B	4
46	B	B	5	B	4
47	A	B	5	B	4
48	C	C	5	C	4
49	E	D	5	D	4
50	A	A	5	A	4
51	B	B	5	B	4
52	B	B	5	B	4
53	D	D	5	D	4
54	C	C	5	C	4
55	C	D	5	B	4
56	E	C	5	C	4
57	D	D	5	D	4
58	A	A	5	A	4
59	E	E	5	E	4
60	E	C	5	E	4
61	A	A	5	A	5
62	D	A	5	A	4
63	B	B	5	D	4
64	C	C	5	C	5
65	E	E	5	E	4
66	E	E	5	E	4
67	B	B	5	A	4
68	E	E	5	E	4
69	A	A	5	A	4
70	B	B	5	B	4
71	E	E	5	E	4
72	E	E	5	E	4
73	C	E	5	C	4
74	E	E	5	A	4
75	C	C	5	C	5
76	B	B	5	B	4
77	B	B	5	B	4
78	C	C	5	C	5
79	E	E	5	C	4
80	C	C	5	C	4
81	E	E	5	E	4
82	C	C	5	C	5
83	B	B	5	B	4
84	E	E	5	E	4
85	D	D	5	D	4
86	E	E	5	E	4
87	D	D	5	D	4
88	E	E	5	E	4
89	D	D	5	D	4
90	D	D	5	D	4
91	A	E	5	E	4
92	D	D	5	D	5
93	E	D	5	D	4
94	B	B	5	B	4
95	E	E	5	E	4
96	A	D	5	A	4
97	D	B	5	D	4
98	C	C	5	C	5
99	C	E	5	C	4
100	C	C	5	C	4
101	C	C	5	C	4
102	D	D	5	D	4
103	B	B	5	B	4
104	E	B	5	B	4
105	E	D	5	D	4
106	A	A	5	A	5
107	E	E	5	E	4
108	D	C	5	C	4
109	E	E	5	E	4
110	D	D	5	D	4
111	B	B	5	B	4
112	Question withdrawn				
113	A	A	5	A	4
114	E	D	5	B	4
115	A	A	5	A	4
116	B	E	5	E	4
117	E	D	5	D	4
118	C	B	5	B	4
119	A	A	5	A	5
120	E	E	5	E	4

Taking into account the classification of questions into ‘clinical cases’ and 'other’ GPT-4o answered 18 ‘clinical cases’ questions correctly (69.23%) and eight incorrectly (30.77%) and 67 ‘other’ questions correctly (72.05%) and 26 incorrectly (27.96%) with p-value 0.972 which suggests that there is no statistically significant difference between the responses to 'clinical cases’ and 'other' questions in terms of correct and incorrect answers for GPT-4o. DeepSeek-R1 answered 17 ‘clinical cases’ questions correctly (65.38%) and nine incorrectly (34.62%) and 71 ‘other’ questions correctly (76.34%) and 22 incorrectly (23.66%) with p-value 0.383 which, as in the case of the GPT-4o model, also indicates that there is no statistically significant difference in the distribution of correct and incorrect responses between 'clinical cases' and 'other' question types (Table 2).

**Table 2:** GPT-4o and DeepSeek-R1 results in “clinical cases” and “other” questions. In the statistical analysis, the chi-square test was used. A result was considered statistically significant if p < 0.05.

ChatGPT version	Correct answers n (%)	Incorrect answers n (%)	p-value	χ2-value
ChatGPT 4.o				
Clinical cases	18 (69.23)	8 (30.77)	p = 0.972	χ2=0.0012
Other	67 (72.04)	26 (27.96)		
DeepSeek				
Clinical cases	17 (65.38)	9 (34.62)	p = 0.383	χ2=0.7619
Other	71 (76.34)	22 (23.66)		

**Figure 2:**
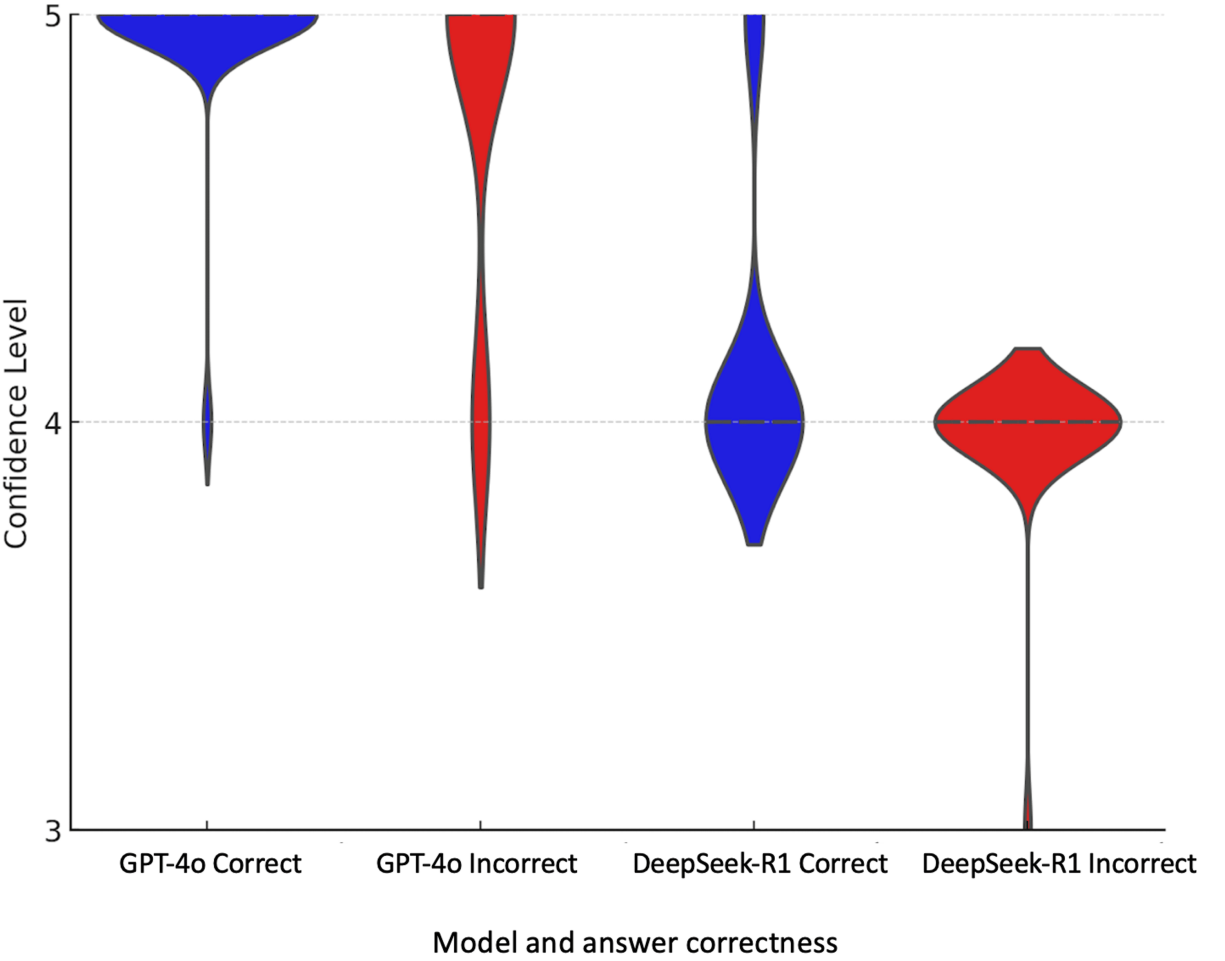
Comparison of confidence levels for correct and incorrect answers for GPT-4o and DeepSeek-R1. In the statistical analysis of the distribution of correct and incorrect responses for both AI models, the Mann-Whitney test was used. A result was considered statistically significant if p < 0.05. GPT-4o The p-value is 0.0026, indicating a statistically significant difference between the confidence distributions for correct and incorrect answers. This difference may suggest that GPT-4o is more confident in its correct answers compared to its incorrect ones. Deep-Seek-R1 The p-value is 0.0026, indicating a statistically significant difference between the confidence distributions for correct and incorrect answers. This difference may suggest, similarly to GPT-4o, that Deep-Seek-R1 is more confident in its correct answers compared to its incorrect ones.

In the Mann-Whitney test analysing confidence levels in both correct and incorrect responses, GPT-4o yielded a p-value of 0.0026, and DeepSeek-R1 yielded a p-value of 0.0076, indicating that for both AI models, there is a statistically significant difference between the distributions of confidence for correct and incorrect answers (Figure 2).

## Discussion

The application of artificial intelligence in medicine has made significant progress in recent years [17]. The impact of artificial intelligence in healthcare is evident across a wide range of applications, including the detection of clinical conditions through medical imaging and diagnostic services, early diagnosis and management of the COVID-19 pandemic, delivery of virtual patient care via AI-powered tools, efficient management of electronic health records, enhancement of patient engagement and adherence to treatment plans, reduction of administrative burdens on healthcare professionals, facilitation of drug and vaccine discovery, identification of prescription errors, advanced data storage and analytical capabilities, as well as technology-assisted rehabilitation [18]. These findings align with a growing body of work exploring novel educational tools in medicine, such as spaced repetition systems developed by Koenig et al to enhance learning in plastic surgery and other specialties [19]. The COVID-19 pandemic has underscored the critical importance of knowledge in the field of infectious diseases, which is the focus of the present study.

During the Autumn 2024 session of the Polish specialty examination in infectious diseases, 16 candidates took the exam. The average score obtained by the examinees was 84.2 points (70.76%). The passing threshold was set at 72 points (60.5%). Three candidates (18.75%) did not meet the required threshold and consequently received a failing result. The median score was 84 points (70.59%), with the highest score being 100 points (84.03%) and the lowest score being 66 points (55.46%) [20]. Unlike medical students and physicians taking the LEK, physicians sitting for the specialization examination do not have prior access to a question bank. In the case of the LEK, this bank currently comprises approximately 70% of the questions appearing on each exam. The questions used in Polish specialization examinations are entirely new; however, before taking the exam, candidates have access to questions from previous years, which they may use to assess their knowledge and assist in preparation. Nonetheless, they cannot expect identical questions to appear on the specialization examination [9]. Unlike medical students and recent graduates taking the LEK, physicians undertaking specialization examinations have typically completed five years of daily clinical practice with patients-in the case of infectious diseases-providing them with a broader and more in-depth knowledge base, as well as the ability to apply this knowledge in a practical context within their chosen field of interest. In contrast, LEK candidates are examined across all areas of medicine, regardless of their individual interests, and generally lack substantial clinical experience [9,10,21].

The results obtained in the study indicate that both GPT-4o and DeepSeek-R1 would have successfully passed the specialization examination in infectious diseases, exceeding the 72 (60,5%) passing threshold by 13 (10.92%) and 16 (13.44%), respectively. The percentage scores achieved by both AI models are comparable to the average score attained by human examinees, which was 84.2 (70.76%). Specifically, GPT-4o outperformed the average by 0.8 (0.67%), while DeepSeek-R1 exceeded it by 3.8 (3.19%).

The difference in performance between the tested AI models amounted to 3 (2.52%) in favour of DeepSeek-R1. Considering the significantly shorter period of operation of this model, as well as the substantial differences in the financial resources invested and the computational power required for its operation, this result draws particular attention to DeepSeek-R1 and highlights the potential for future AI model development with considerably lower financial and hardware demands [6].

An additional important finding emerging from the study is the absence of a statistically significant difference in the performance of the AI models when responding to clinical case questions versus other questions related to infectious diseases that were not classified as clinical scenarios. This suggests that both models demonstrate a comparable capacity for analysing complex clinical cases and addressing non-case-based content within the same medical specialty. This result holds particular significance in the context of potential future applications of AI in clinical practice, where patient data could be entered into an AI system and the generated output might provide valuable support for physicians in enhancing diagnostic precision and therapeutic decision-making. However, the current level of performance is not yet sufficient to justify full reliance on AI models for autonomous diagnosis or treatment. Therefore, continued development of these technologies, as well as careful monitoring of future AI models and rigorous research into their applicability in real-world clinical settings, remains essential. 

It is also important to acknowledge the limitations of the study, which include the relatively small number of questions used, the limited number of candidates who took the examination during the analyzed session - factors that reduced the reliability of statistical analysis - and the use of the Polish language when presenting questions to both AI models.

## Conclusions

The results obtained by GPT-4o and DeepSeek-R1 indicate that the examined AI models may hold significant potential as educational tools and, in the future, given their continued development, as important instruments supporting physicians in accurate diagnosis and effective patient treatment.

The relatively small differences in effectiveness between the two AI models - despite substantial disparities in their duration of operation, development costs, and required computational resources - may suggest that future AI models could be further improved with significantly lower financial and hardware investments, while still achieving enhanced performance.
